# The δ-Opioid Receptor Differentially Regulates MAPKs and Anti-inflammatory Cytokines in Rat Kidney Epithelial Cells Under Hypoxia

**DOI:** 10.3389/fphys.2019.01572

**Published:** 2020-01-21

**Authors:** Fengbao Luo, Renfang Xu, Guanglai Song, Hao Lu, Xiaozhou He, Ying Xia

**Affiliations:** ^1^Department of Urology, The Third Affiliated Hospital of Soochow University, Changzhou, China; ^2^Shanghai Key Laboratory of Acupuncture Mechanism and Acupoint Function, Fudan University, Shanghai, China

**Keywords:** kidney, NRK-52E cell, hypoxia, DOR, MAPKs, Bcl-6

## Abstract

Hypoxic injury is one of the most important factors in progressive kidney disorders. Since we have found that δ-opioid receptor (DOR) is neuroprotective against hypoxic stress through a differential regulation of mitogen-activated protein kinases (MAPKs) and anti-inflammatory cytokines, we asked if DOR that is highly expressed in the kidney can modulate renal MAPKs and anti-inflammatory cytokines under hypoxia. We exposed cultured rat kidney epithelial cells (NRK-52E) to prolonged hypoxia (1% O_2_) with applications of specific DOR agonist or/and antagonist to examine if DOR affects hypoxia-induced changes in MAPKs and anti-inflammatory cytokines. The results showed that endogenous DOR expression remained unchanged under hypoxia, while DOR activation with UFP-512 (a specific DOR agonist) reversed the hypoxia-induced up-regulation of ERK1/2 and p38 phosphorylation. DOR inhibition with naltrindole had no appreciable effect on the hypoxia-induced changes in ERK1/2 phosphorylation, but increased p38 phosphorylation. DOR inhibition with naltrindole attenuated the effects of DOR activation on the changes in ERK1/2 and p38 phosphorylation in hypoxia. Moreover, DOR activation/inhibition differentially affected the expression of transcriptional repressor B-cell lymphoma 6 (Bcl-6), anti-inflammatory cytokines tristetraprolin (TTP), and interleukin-10 (IL-10). Taken together, our novel data suggest that DOR activation differentially regulates ERK1/2, p38, Bcl-6, TTP, and IL-10 in the renal cells under hypoxia.

## Introduction

Alteration in oxygenation induced tissue hypoxia in kidney has been identified as a final common pathway to the development and progression of diseases in both acute and chronic kidney injury (Nangaku et al., 2013; Singh et al., 2013; Fu et al., 2016), as well as renal cancer (Myszczyszyn et al., 2015; Schodel et al., 2016), and eventually leads to the end-stage kidney disease (ESKD). The responses of individual cells to hypoxia include a range of changes in genetic and signaling regulations that facilitate them to adapt to the hypoxic conditions (Shoji et al., 2014). It is demonstrated that multiple signaling pathways, such as hypoxia inducible factor (HIF), Notch, NF-κB, PI3K/Akt, and mitogen-activated protein kinases (MAPKs), are involved in hypoxia response (Luo et al., 2016; Liu J. et al., 2017). The MAPKs are one of the most fascinating signaling pathways that have been frequently studied and involved in many hypoxic/ischemic kidney diseases (Kim et al., 2013; Liu et al., 2013).

In MAPK family, there are at least four members based on their sequence similarity, upstream activators, and to a lesser extent, substrate specificity (Kyriakis and Avruch, 2001). The classic extracellular signal-regulated kinases1 and 2 (ERK1 and ERK2) are identified in the context of growth factor-related signaling, whereas the c-Jun N-terminal kinase (JNK) and p38 families are described in the setting of cell response to stress and inflammation (Kyriakis and Avruch, 2012). All MAPK pathways include central three-tiered “core signaling modules” as MAPK-kinase-kinase, in which MAPKs are activated by associated Tyr and Thr phosphorylation (Kyriakis and Avruch, 2001). They are differentially involved in the regulation of inflammatory cytokines in the hypoxic/ischemic conditions (Tan et al., 2013; Wang et al., 2017; Wu et al., 2017).

Mitogen-activated protein kinases also participate in renal responses to hypoxic stress. The activation of MAPKs, especially ERK1/2 and p38, is detected under hypoxic condition in human kidney (Chen et al., 2013). Our recent studies on rat kidney epithelial cells have shown that the phosphorylation of both ERK1/2 and p38 is up-regulated under hypoxic stress (Luo et al., 2017). However, this is different from our observations on neuronal cells that showed an increase in p38 phosphorylation with a decrease in the level of ERK1/2 phosphorylation after prolonged exposure to severe hypoxia (Ma et al., 2005).

Our serial studies have proven that the δ-opioid receptor (DOR) has a protective effect on neurons under hypoxic/ischemic stress via the regulation of ERK1/2 and p38 as well as inflammatory cytokines (Ma et al., 2005). DOR is a G protein-coupled receptor and is a key player in pain control, hedonic homeostasis, mood, stress responses, and other function (Chu Sin Chung and Kieffer, 2013; Xia, 2015). Since DOR is highly expressed in the kidney (Peng et al., 2012), we wonder if DOR activation modulates survival process by interacting with MAPKs as well as anti-inflammatory cytokines such as transcriptional repressor B-cell lymphoma 6 (Bcl-6), anti-inflammatory cytokines tristetraprolin (TTP), and interleukin-10 (IL-10) in hypoxic renal cells. Based on our previous studies, we hypothesize that DOR activation may differentially regulate MAPKs and some anti-inflammatory cytokines in the kidney under hypoxia. To test this hypothesis, we conduct serial experiments on rat kidney epithelial cells in this work to determine DOR expression and the effects of DOR activation or/and inhibition on ERK1/2, p38, and some critical anti-inflammatory cytokines under prolonged hypoxia. The outcome results demonstrate that DOR signaling indeed differentially regulates ERK1/2, p38, Bcl-6, TTP, and IL-10 under hypoxic conditions.

## Materials and Methods

### Cell Culture

NRK-52E cells (normal rat kidney epithelial cells) were acquired from Chinese Academy of Sciences (Shanghai, China). In brief, Dulbecco’s Modified Eagle Medium (DMEM) (Gibco, Grand Island, NY, United States) was used as cell culture medium, containing 5% fetal bovine serum (FBS) (Gibco, Grand Island, NY, United States), 100 U/ml penicillin, and 100 mg/ml streptomycin (Gibco, Grand Island, NY, United States). NRK-52E cells were incubated in a humidified atmosphere at 37°C with 21% O_2_ and 5% CO_2_.

### Establishment of Hypoxic Culture Condition

Cells were exposed to hypoxia by placing them in a mixed gas incubator filled with an atmosphere consisting of 94% N_2_, 5% CO_2_, and 1% O_2_ for different durations (24, 48, and 72 h). Normoxic controls were incubated in parallel in a humidified incubator supplemented with 21% O_2_ and 5% CO_2_ at 37°C.

### Drug Treatments

For drug treatments, UFP-512, a specific and effective DOR agonist, was synthesized by our research teams (Balboni et al., 2002; Chao et al., 2007), naltrindole, a particular DOR antagonist (Zhang et al., 2006; Granier et al., 2012), was purchased from Sigma (St. Louis, MO, United States), tumor necrosis factor-α (TNF-α) was purchased from R&D Systems (Minneapolis, MN, United States), they were added into the culture medium in demanded concentrations and exposed to normoxic or hypoxic conditions starting immediately before the onset of hypoxia (or at the same time-point in normoxia).

### Cellular Proliferation Assay

The NRK-52E cells (1 × 10^4^ cells/well) were seeded in a 96-well plate and incubated in normal culture condition for 24 h before hypoxia. After drug treatment, cell proliferation was determined by the CellTiter 96^®^ AQueous One Solution Cell Proliferation Assay (MTS) kit (Promega, Madison, WI, United States) according to the manufacturer’s instructions. MTS reagent (10 μl) was added to cultured cells in all wells followed by incubation for 1 h at 37°C. The absorbance was tested at the wavelength of 490 nm with a micro-plate reader (Bio-Rad Laboratories, Hercules, CA, United States).

### Protein Extraction and Western Blot Analysis

Harvested cells were washed twice with ice-cold phosphate-buffered saline (PBS) and homogenized with lysis buffer (KeyGEN Biotech Co., Ltd., Nanjing, China) on ice. Cell lysates were centrifuged at 4°C for 15 min at 12,000 rpm, and then the supernatant was collected in fresh tubes and quantified using the bicinchoninic acid assay (BCA) method (KeyGEN Biotech Co., Ltd., Nanjing, China). Cell lysate proteins (30 μg) were separated by sodium dodecyl sulfate-polyacrylamide (Solarbio Science & Technology Co., Ltd., Beijing, China) gel electrophoresis and electrophoretically transferred to polyvinylidene difluoride (PVDF) membranes (Millipore, Billerica, MA, United States). After blocking with 5% fat-free milk (Bio-Rad Laboratories, Hercules, CA, United States) in Tris-buffered saline tween-20 (TBST, Solarbio Science & Technology Co., Ltd., Beijing, China) buffer at room temperature for 1 h, the membranes were incubated overnight at 4°C with the indicated primary antibodies. β-Actin (1:3000), ERK1/2 (1:2000), and p38 (1:1000) were purchased from Cell Signaling Technologies (Danvers, MA, United States), rabbit polyclonal anti-DOR (1:1000) were from Millipore (Billerica, MA, United States), Bcl-6 (1:100) and TTP (1:200) were purchased from Santa Cruz Biotechnology (Santa Cruz, CA, United States), IL-10 (1:1000) were purchased from Abcam (Cambridge, United Kingdom). After repeated washing, they were reacted with the appropriate horseradish peroxidase (HRP)-conjugated secondary antibodies for 1 h at room temperature and enhanced chemiluminescence (ECL) detecting reagent (Thermo Scientific, Rockford, IL, United States). The images were analyzed with Quantity One software (Bio-Rad Laboratories, Hercules, CA, United States).

### Statistics

The data were expressed as the changes in percentage compared to the control. At least three independent experiments were conducted in each group. The results were stated as the mean and standard error mean (SEM). Comparison between two groups (i.e., 24 h of normoxia vs. 24 h of hypoxia) was analyzed by unpaired *t*-test. Differences with a *P*-value < 0.05 were considered statistically significant. GraphPad Prism software (GraphPad Software, Inc., La Jolla, CA, United States) was used for statistical analyses.

## Results

### Effect of Hypoxia on the Expression of DOR

**FIGURE 1:**
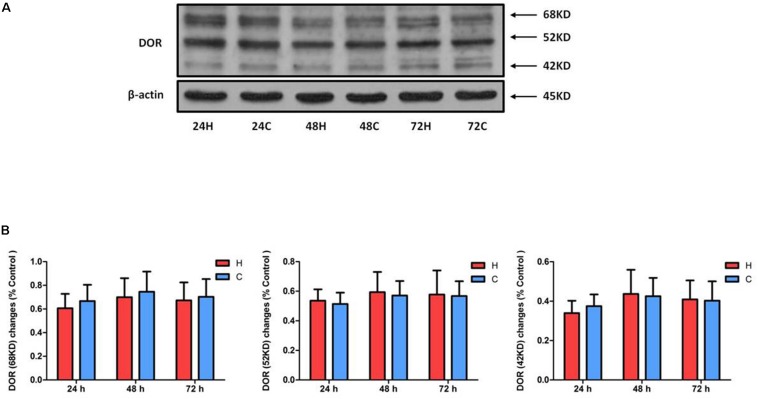
Effect of hypoxia on DOR expression of NRK-52E cells. NRK-52E cells were exposed to hypoxia at 1% O_2_ for 24–72 h. Representative Western blots **(A)** and relative quantitation **(B)** of DOR in the total protein of NRK-52 cells. H, hypoxia; C, normoxic control. At least three independent experiments were conducted in each group. Note that the DOR protein has three different signal bands (68, 52, and 42 KD) and did not change significantly in response to hypoxia at the whole cell level.

We first examined DOR expression in NRK-52E cells under hypoxic conditions (1% O_2_). As shown in Figure 1, hypoxia had no significant impact on the level of DOR protein (68, 52, and 42 KD) after 24–72 h of hypoxia (*P* > 0.05), suggesting that DOR protein expression of NRK-52E cells is resistant to prolonged hypoxic stress, which is very different from the observation made on neuronal cells in our previous study (Ma et al., 2005).

### Effect of DOR Activation on ERK1/2 and p38 in Hypoxic Conditions

**FIGURE 2:**
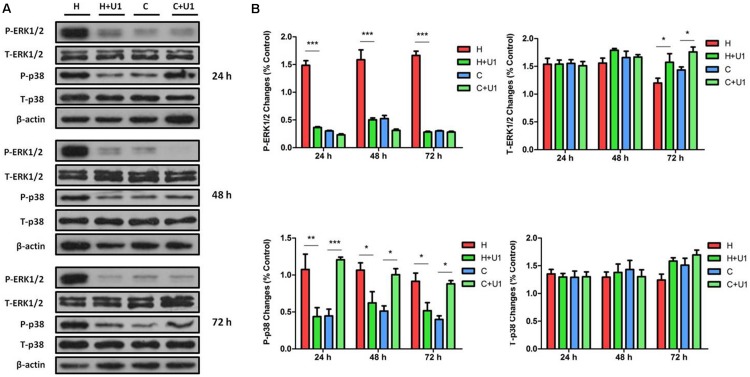
ERK1/2 and p38 expression in NRK-52E cells under hypoxic condition with DOR activation. NRK-52E cells were exposed to hypoxia at 1% O_2_ for 24–72 h and treated with 1 μM DOR agonist (UFP-512). **(A)** Representative Western blots of ERK1/2 and p38 protein. **(B)** Relative quantitation of ERK1/2 and p38. H, hypoxia; C, normoxic control. ^∗^*P* < 0.05, ^∗∗^*P* < 0.01, ^∗∗∗^*P* < 0.001. U1, 1 μM UFP-512; C + U1, DOR activation with 1 μM UFP-512 treatment under normoxic conditions; H + U1, DOR activation with 1 μM UFP-512 treatment under hypoxic conditions. At least three independent experiments were carried out in all groups. Note that DOR activation with UFP-512 eliminated the hypoxia-induced changes on P-ERK1/2 and P-p38.

Our previous work showed that prolonged hypoxia can dramatically induce the activation of MAPKs (ERK1/2 and p38) in NRK-52E cells (Luo et al., 2017), while we also explored that MAPKs were managed by DOR function in neuronal cells under hypoxic tensions (Ma et al., 2005). To explore potential interactions between DOR and MAPKs in rat kidney epithelial cells under hypoxic conditions, we applied UFP-512 (1 μM), a potent and specific agonist of DOR (Balboni et al., 2002; Chao et al., 2008), to the culture medium and determined the effect of DOR activation on ERK1/2 and p38 phosphorylation. After 24–72 h of exposure to UFP-512 in hypoxic conditions, the total cellular content of phosphorylated ERK1/2 (P-ERK1/2) was significantly decreased to 24.73 ± 3.3, 31.99 ± 3.2, and 16.97 ± 2.05%, respectively, compared to those of hypoxic groups without UFP-512 treatment (100%, *P* < 0.001 at all three time points, Figure 2). Interestingly, UFP-512 did not change the ERK1/2 phosphorylation in the same cells under normoxic conditions. In sharp contrast to the changes in P-ERK1/2, the total ERK1/2 (T-ERK1/2) had no appreciable change after DOR activation in both hypoxic groups and normoxic groups after 24 and 48 h of exposure, though UFP-512 induced significant increase of T-ERK1/2 in both hypoxic groups (1.31-fold, *P* < 0.05) and normoxic groups (1.22-fold, *P* < 0.05) after 72 h of treatment (Figure 2). This data suggest that DOR activation can reverse the increase in ERK1/2 phosphorylation induced by hypoxia in rat kidney epithelial cells.

When DOR was activated, the hypoxic increase in phosphorylated p38 (P-p38) was reduced to 39.07 ± 7.01, 59.66 ± 25.65, and 55.45 ± 10.31% after 24, 48, and 72 h, respectively, compared to those of hypoxic groups without UFP-512 treatment (100%, *P* < 0.01 at 24 h and *P* < 0.05 at 48 and 72 h). In sharp contrast, P-p38 was markedly increased by 3.05-, 2.0-, and 2.3-fold at 24, 48, and 72 h, respectively, with UFP-512 treatment in the cells under normoxic conditions (Figure 2). DOR activation had no appreciable effect on the levels of total p38 protein (T-p38) under hypoxia or normoxia. This data suggest that DOR activation can largely reverse the effect of hypoxia on ERK1/2 and p38 phosphorylation. Meanwhile, UFP-512 significantly induced P-p38, but not P-ERK1/2, under normoxic conditions.

### Effects of DOR Inhibition on ERK1/2 and p38 in Hypoxic Conditions

**FIGURE 3:**
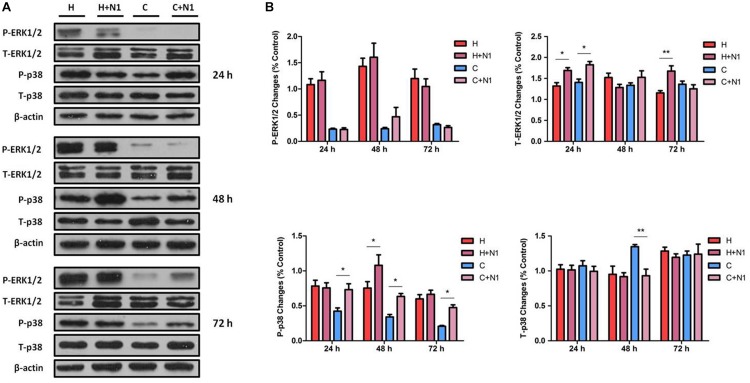
ERK1/2 and p38 expression in NRK-52E cells under hypoxic condition with DOR inhibition. Cells were exposed to hypoxia at 1% O_2_ for 24–72 h and treated with 1 μM DOR antagonist naltrindole. **(A)** Representative Western blots of ERK1/2 and p38 protein. **(B)** Relative quantitation of ERK1/2 and p38. H, hypoxia; C, normoxic control. ^∗^*P* < 0.05, ^∗∗^*P* < 0.01. N1, 1 μM naltrindole; C + N1, DOR inhibition with 1 μM naltrindole treatment under normoxic conditions; H + N1, DOR inhibition with 1 μM naltrindole treatment under hypoxic conditions. At least three independent experiments were performed. Note that DOR-inhibition with naltrindole had no obvious impact on the expression of P-ERK1/2 under either hypoxia or normoxia, whereas naltrindole can increase the T-ERK1/2 after 24 h of treatment using both hypoxia and normoxia, as well as after 72 h of treatment in the hypoxic group. The level of P-p38 was highly induced by the naltrindole treatment under normoxic and hypoxic condition (48 h), though the level of T-p38 only changed significantly in response to naltrindole at 48 h of treatment under normoxia.

To further verify the roles of DOR in MAPK regulation in NRK-52E cells exposed to hypoxic stress, we tested the effects of naltrindole, a specific DOR antagonist (Zhang et al., 2006; Filizola and Devi, 2012; Granier et al., 2012), on the changes in ERK1/2 and p38 phosphorylation. As shown in Figure 3, incubation with naltrindole (1 μM) alone had no significant impact on ERK1/2 phosphorylation under normoxia or hypoxia (*P* > 0.05). However, it resulted in a significant increase in T-ERK1/2 expression in both hypoxic (1.28-fold, *P* < 0.05) and normoxic (1.30-fold, *P* < 0.05) conditions at 24 h, as well as 72 h after hypoxia (1.45-fold, *P* < 0.05).

The effect of DOR inhibition on p38 was different from that of ERK1/2. As shown in Figure 3, DOR inhibition by 1 μM naltrindole significantly up-regulated the levels of P-p38 by 1.73-, 1.86-, and 2.29-fold at 24, 48, and 72 h, respectively, under normoxic conditions (*P* < 0.05). In the hypoxic groups, naltrindole increased p38 phosphorylation after 48 h, but not 24 or 72 h, of the drug treatment (1.43-fold, *P* < 0.05, Figure 3). The expression of T-p38 had no appreciable change after naltrindole administration except a large reduction at 48 h after hypoxia (decreased to 69.45 ± 14.71%, *P* < 0.01, Figure 3).

### Attenuated Effect of DOR Antagonist on DOR Activation Induced Changes in ERK1/2 and p38

**FIGURE 4:**
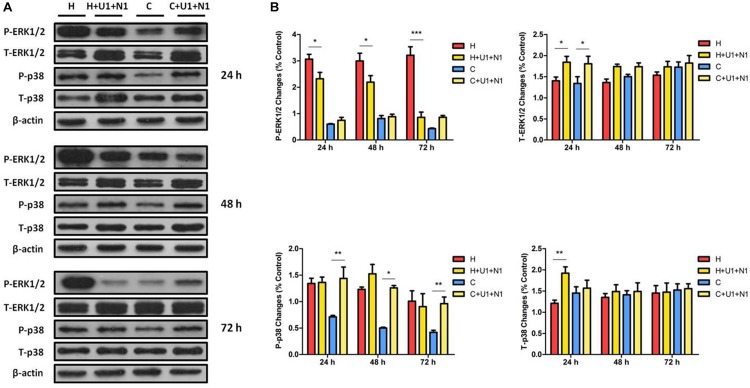
The effects of DOR activation and inhibition on ERK1/2 and p38 in NRK-52E cells. NRK-52E cells were exposed to hypoxia with both DOR agonist UFP-512 (1 μM) and DOR antagonist naltrindole (1 μM) at 1% O_2_ for 24–72 h. **(A)** Representative Western blots of ERK1/2 and p38 protein. **(B)** Relative quantitation of ERK1/2 and p38. H, hypoxia; C, normoxic control. ^∗^*P* < 0.05, ^∗∗^*P* < 0.01, ^∗∗∗^*P* < 0.001. U1 + N1, simultaneous administration of 1 μM UFP-512 and 1 μM naltrindole; C + U1 + N1, 1 μM UFP-512 and 1 μM naltrindole treatment under normoxic conditions; and H + U1 + N1, 1 μM UFP-512 and 1 μM naltrindole treatment under hypoxic conditions. At least three independent experiments were conducted in each group. Note that naltrindole, along with UFP-512, reversed the efforts on ERK1/2 phosphorylation induced by hypoxia, especially after 72 h of treatment, whereas both the DOR agonist and antagonist induced the expression of T-ERK1/2 significantly after exposure for 24 h. The P-p38 was largely increased by naltrindole and UFP-512 treatment under normoxia, while the T-p38 protein levels had markedly increased under hypoxia after 24 h of treatment.

We further investigated if DOR antagonist can mitigate the DOR activation induced effects stated above. When simultaneously treating the hypoxic cells with both UFP-512 (1 μM) and naltrindole (1 μM), the level of P-ERK1/2 was reduced to 75.38 ± 6.49% at 24 h (*P* < 0.05), 75.06 ± 22.37% at 48 h (*P* < 0.05), and 28.37 ± 14.13% at 72 h (*P* < 0.001), compared to those of hypoxia alone. In normoxic cells, the treatment did not change the P-ERK1/2 level at all. The co-treatment of UFP-512 and naltrindole elevated the levels of T-ERK1/2 in both hypoxic and normoxic groups, with a more remarkable increase after the first 24 h (*P* < 0.05, Figure 4). These observations suggest that DOR activation with UFP-512 can significantly reduce ERK1/2 phosphorylation and this effect was partly attenuated by co-exposure to naltrindole, although naltrindole treatment alone had no impact on the levels of P-ERK1/2.

P-p38 was dramatically increased by 2.02-, 2.51-, and 2.29-fold at 24, 48, and 72 h with the co-treatment of UFP-512 and naltrindole in the cells under normoxic conditions. The same treatment also increased T-p38 expression under hypoxia, but only occurred at 24 h (1.59-fold, *P* < 0.01, Figure 4). This data suggested that DOR activation with UFP-512 can remarkably decrease p38 phosphorylation under hypoxia though it increased p38 phosphorylation under normoxia, while naltrindole can attenuate the UFP-512-induced decrease in p38 phosphorylation under hypoxia.

### Changes in ERK1/2 and p38 Phosphorylation in Response to TNF-α Exposure

**FIGURE 5:**
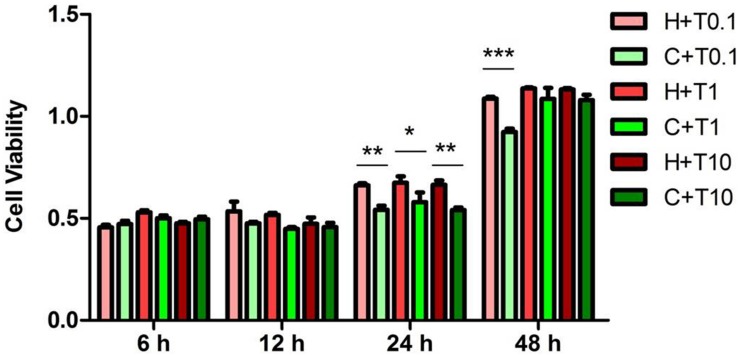
Effects of TNF-α treatment on the viability of NRK-52E cells exposed to hypoxia. NRK-52E cells were exposed to hypoxia at 1% O_2_ with TNF-α, and then the cell viability (*n* = 4) was measured by an MTS (3-[4,5-dimethylthiazol-2-yl]-5-[3-carboxymethoxypheny]-2-[4-sulfophenyl]-2H-tetrazolium) assay. H, hypoxia; C, normoxic control; T0.1, 0.1 ng/ml TNF-α; T1, 1 ng/ml TNF-α; T10, 10 ng/ml TNF-α. ^∗^*P* < 0.05, ^∗∗^*P* < 0.01, ^∗∗∗^*P* < 0.001. Note that the cell viability treated by TNF-α under hypoxia was higher than under normoxia after 24 and 48 h (0.1 ng/ml TNF-α).

Since the TNF-α has been thought as a pro-inflammatory mediator for hypoxic/ischemic injury (Park and Bowers, 2010; Watters and O’Connor, 2011; Liu and McCullough, 2013; Wang et al., 2014), we further examined the response of ERK1/2 and p38 to TNF-α exposure in the NRK-52E cells. We administered TNF-α to the medium at three increasing concentrations (0.1, 1, and 10 ng/ml) and examined their effects on ERK1/2 and p38 at 6, 12, 24, and 48 h in hypoxia and normoxia (Figure 5). After the first 12 h of TNF-α exposure in either hypoxia or normoxia, the MTS assay showed that the NRK-52E cells did not have any appreciable injury. After prolonging the duration of TNF-α exposure to 24 h, normoxic cells showed severe damage with a significant reduction of cell viability compared to those of normoxic cells without TNF-α treatment. As a comparison, the same treatment did not induce cell injury in hypoxia. After 48 h of exposure to TNF-α at 0.1 ng/ml, cell viability was significantly lower in the normoxic than hypoxic groups (*P* < 0.01). In the concentrations of 1 and 10 ng/ml TNF-α; however, there was no appreciable difference in cell viability between normoxic and hypoxic groups.

**FIGURE 6:**
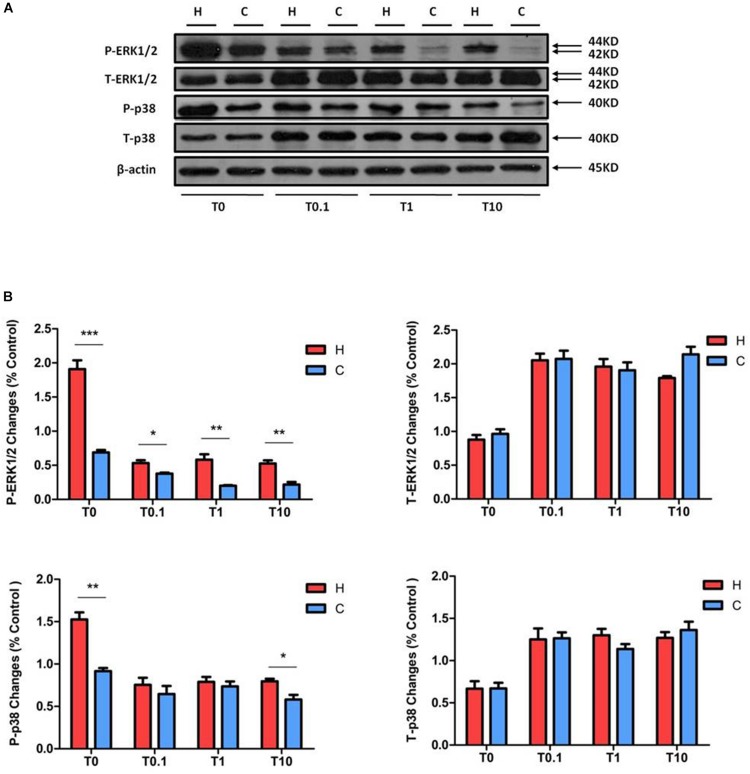
Effects of TNF-α treatment on the expression of ERK1/2 and p38 in NRK-52E cells. Cells were exposed to media with different concentration of TNF-α at 1% O_2_ for 24 h. The expressions of ERK1/2 and p38 were measured by a Western blot. **(A)** Representative Western blots of ERK1/2 and p38 protein. **(B)** Relative quantitation of ERK1/2 and p38. H, hypoxia; C, normoxic control; T0.1, 0.1 ng/ml TNF-α; T1, 1 ng/ml TNF-α; T10, 10 ng/ml TNF-α. ^∗^*P* < 0.05, ^∗∗^*P* < 0.01, ^∗∗∗^*P* < 0.001. At least three independent experiments were performed. The levels of P-ERK1/2 treated with different concentrations of TNF-α were relatively elevated in the groups of hypoxia, compared to the groups treated with TNF-α under normoxia. Similar to P-ERK1/2, P-p38 under hypoxia was higher than under normoxia, but only occurred after treatment with 10 ng/ml TNF-α. TNF-α induced the total protein levels of ERK1/2 and p38; there was no significant change between the groups under hypoxia and normoxia.

After exposure for 24 h to TNF-α with increasing concentrations (0.1, 1, and 10 ng/ml) in hypoxia, P-ERK1/2 was largely increased by hypoxia alone, while TNF-α treatment fully eliminated the hypoxic effect on ERK1/2 phosphorylation. In the condition of TNF-α, the P-ERK1/2 expression under normoxia was much lower than that under hypoxia (decreased to 71.62 ± 9.08% at 0.1 ng/ml TNF-α, *P* < 0.05; 35.49 ± 6.20% at 1 ng/ml TNF-α, *P* < 0.01; and 40.91 ± 6.15% at 10 ng/ml TNF-α, *P* < 0.01, Figure 6). There was a significant increase in T-ERK1/2 levels after TNF-α exposure, with no appreciable difference between hypoxic and normoxic groups.

The P-p38 was decreased after TNF-α treatment under hypoxic conditions, there was no significant change between hypoxic and normoxic groups except after the exposure to 10 ng/ml TNF-α (decreased to 72.73 ± 7.7% in normoxia at 10 ng/ml TNF-α, compared to those of hypoxic groups at 10 ng/ml TNF-α, *P* < 0.05, Figure 6). The T-p38 protein level was significantly enhanced by TNF-α exposure at all concentrations. In short, TNF-α treatment reduced cell viability and the phosphorylation of ERK1/2 and p38 in rat kidney epithelial cells, but increased the expression of T-ERK1/2 and T-p38 protein levels. Interestingly, in all concentrations of TNF-α exposure, the P-ERK1/2 level was significantly elevated in hypoxia compared to that of normoxic group.

### Effect of DOR Activation or/and Inhibition on Bcl-6, TTP, and IL-10

**FIGURE 7:**
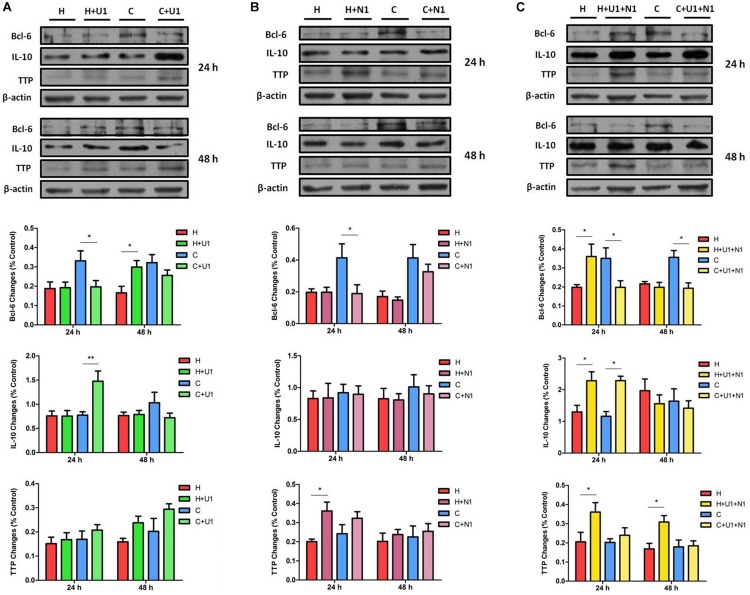
The protein expression of Bcl-6, TTP, and IL-10 in NRK-52E cells under hypoxic condition with DOR activation or/and inhibition. Cells were exposed to hypoxia at 1% O_2_ for 24–48 h and treated with 1 μM DOR agonist (UFP-512) or/and 1 μM DOR antagonist naltrindole. **(A)** Western blots of Bcl-6, TTP, and IL-10 after UFP-512 treatment. **(B)** Western blots of Bcl-6, TTP, and IL-10 after naltrindole treatment. **(C)** Western blots of Bcl-6, TTP, and IL-10 after UFP-512 and naltrindole treatment. H, hypoxia; C, normoxic control. ^∗^*P* < 0.05, ^∗∗^*P* < 0.01. U1, 1 μM UFP-512; C + U1, DOR activation with 1 μM UFP-512 treatment under normoxic conditions; H + U1, DOR activation with 1 μM UFP-512 treatment under hypoxic conditions; N1, 1 μM naltrindole; C + N1, DOR inhibition with 1 μM naltrindole treatment under normoxic conditions. U1 + N1, simultaneous administration of 1 μM UFP-512 and 1 μM naltrindole; C + U1 + N1, 1 μM UFP-512 and 1 μM naltrindole treatment under normoxic conditions; H + U1 + N1, 1 μM UFP-512 and 1 μM naltrindole treatment under hypoxic conditions.

Since hypoxia significantly decreases the levels of Bcl-6 protein in the NRK-52E cells (Luo et al., 2017) and DOR is cytoprotective against hypoxic stress (Ma et al., 2005; Wang et al., 2014; Cao et al., 2015), we further examined if DOR plays a role in Bcl-6 protein expression. We found that DOR activation with 1 μM UFP-512 had no significant effect on Bcl-6 expression after 24 h of hypoxia; however, it down-regulated the level of Bcl-6 protein by 40.39 ± 1.13% under normoxic conditions (*P* < 0.05). At 48 h after adding the DOR agonist, Bcl-6 protein expression increased in the group of hypoxia (1.87-fold, *P* < 0.05), but did not change significantly in normoxia (Figure 7A). Moreover, the level of Bcl-6 protein was much lower than the control after naltrindole treatment for 24 h in normoxic conditions (*P* < 0.05). This reduction almost disappeared at 48 h. The hypoxic cells showed no significant differences in terms of Bcl-6 expression after DOR inhibition at 24 and 48 h (Figure 7B). In addition, Bcl-6 expression decreased after co-exposure of UFP-512 and naltrindole under normoxia both at 24 and 48 h (*P* < 0.05) and increased at 24 h under hypoxia (*P* < 0.05, Figure 7C). As shown in Table 1, it seems that the expression level of Bcl-6 is linked to MAPKs phosphorylation level, which can be managed by DOR function in rat kidney epithelial cells.

**TABLE 1:** Effects of DOR activation/inhibition on ERK1/2, p38, Bcl-6, TTP, and IL-10.

**Treatment**	**Condition**	**P**-**ERK1/2**	**P**-**p38**	**Bcl**-**6**	**TTP**	**IL**-**10**
DOR activation with UFP-512	Hypoxia	↓(24–72 h)	↓(24–72 h)	↑(48 h)	–	–
	Normoxia	–	↑(24 h), ↑(48, 72 h)	↓(24 h)	–	↑(48 h)
DOR inhibition with Naltrindole	Hypoxia	–	↑(48 h)	–	↑(24 h)	–
	Normoxia	–	↑(24–72 h)	↓(24 h)	–	–
UFP-512 plus Naltrindole	Hypoxia	↓(24, 48 h), ↓(72 h)	–	↑(24 h)	↑(24, 48 h)	↑(24 h)
	Normoxia	–	↑(24, 72 h), ↑(48 h)	↓(24, 48 h)	–	↑(24 h)

*↑, increased and *P* < 0.05; ↑, increased and *P* < 0.01; ↑, increased and *P* < 0.001; ↓, decreased and *P* < 0.05; ↓, decreased and *P* < 0.001.*

As a comparison, we also investigated the effects of DOR activation on TTP protein in the same cells. In normoxia, UFP-512 treatment did not significantly affect the expression of TTP at 24 h after the treatment, but intended to increase TTP protein levels at 48 h (Figure 7A). After naltrindole treatment, the expression of TTP seemed to increase under both normoxia and hypoxia at 24 h, though the phenotype was more significant under hypoxia (Figure 7B, *P* < 0.05). TTP was significantly increased by co-treatment of UFP-512 and naltrindole under hypoxic conditions, but did not change under normoxia (Figure 7C). Since the expression level of TTP remains steady under hypoxic stress, indicated in our previous (Luo et al., 2017), it is reasonable to believe that TTP is also regulated by DOR function in NRK-52E cells.

On the other hand, IL-10 protein level was significantly increased after exposure to UFP-512 for 24 h in normoxic conditions (*P* < 0.01), but not in hypoxic conditions. The increased IL-10 in normoxic conditions disappeared after treatment of UFP-512 for 48 h (Figure 7A). Meanwhile, IL-10 had no response to the DOR inhibition by naltrindole under either hypoxia or normoxia (Figure 7B). Interestingly, the co-treatment of UFP-512 and naltrindole for 24 h can enhance the expression of IL-10 in both normoxic and hypoxic conditions (*P* < 0.05), but not for those of 48 h of treatment (Figure 7C). Similar to TTP, the expression level of IL-10 may be conducted by the DOR function, since hypoxia alone has no appreciable impact on IL-10 in NRK-52E cells (Luo et al., 2017).

### Changes in Bcl-6, TTP, and IL-10 Expression in Response to TNF-α Exposure

**FIGURE 8:**
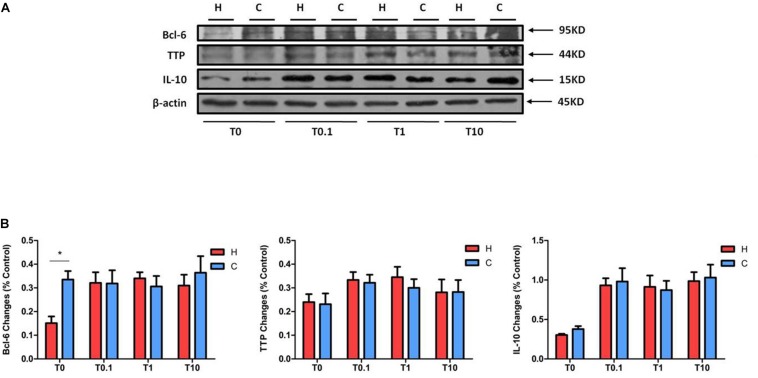
The expression of Bcl-6, TTP, and IL-10 in NRK-52E cells after TNF-α treatment. NRK-52E cells were exposed to media with different concentrations of TNF-α at 1% O_2_ for 24 h. The expressions of Bcl-6, TTP, and IL-10 were measured by Western blot. **(A)** Representative Western blots of Bcl-6, TTP, and IL-10 protein. **(B)** Relative quantitation of Bcl-6, TTP, and IL-10 protein (*n* = 3). H, hypoxia; C, normoxic control; T0.1, 0.1 ng/ml TNF-α; T1, 1 ng/ml TNF-α; T10, 10 ng/ml TNF-α. ^∗^*P* < 0.05. Note that TNF-α can raise the protein levels of Bcl-6 under hypoxia, without changing them between hypoxia and normoxia. TNF-α tended to promote the expression of TTP and IL-10 under both hypoxia and normoxia, though there was no change between the groups under hypoxia and normoxia treated by TNF-α.

We also checked the expressions of Bcl-6, TTP, and IL-10 in NRK-52E cells exposed to TNF-α under hypoxia. Compared to the hypoxic groups without TNF-α treatment, the protein levels of Bcl-6 in the groups with TNF-α were much higher. However, there was no significant difference in Bcl-6 levels between the hypoxic and normoxic groups when disregarding the concentration of TNF-α (Figure 8). The expression of TTP and IL-10, especially the latter, seemed to be higher after TNF-α treatment under both hypoxic and normoxic conditions.

## Discussion

This work presents the following key findings in rat kidney epithelial cells: (1) prolonged hypoxia increased the phosphorylation of ERK1/2 and p38 without any appreciable change in DOR expression, while DOR activation attenuate the hypoxia-induced phosphorylation of ERK1/2 and p38; (2) DOR inhibition with naltrindole had no significant impact on the change of P-ERK1/2 either under hypoxia or normoxia, but increased the phosphorylation of p38 after 48 h of hypoxia and 24–72 h of normoxia; (3) either DOR agonist or antagonist increased the activation of p38 under normoxic conditions, while DOR inhibition with naltrindole partly counteract the effect of DOR activation on the changes of ERK1/2 and p38 phosphorylation under hypoxic conditions; (4) TNF-α decreased the expression of P-ERK1/2; (5) the expression of Bcl-6 was negatively regulated by ERK1/2 and p38 phosphorylation, which could be controlled by DOR function; and (6) the expression of TTP and IL-10 was regulated by DOR administration though hypoxia alone had no significant effect on these two anti-inflammatory cytokines.

The delta-, mu-, and kappa-opioid receptors (DOR, MOR, and KOR) are three major subtypes of opioid receptors and belong to members of the rhodopsin subfamily in the superfamily of seven transmembrane nucleotide binding regulatory G-protein-coupled receptors (Law and Loh, 1999; Audet et al., 2008; Xia, 2015). A current study using absolute quantitative real-time illustrated that the kidney is principally a “DOR organ” since DOR expression is approximately 44.5-fold higher than that of KOR, without any detectable expression of MOR (Peng et al., 2012). Indeed, our studies (Chen et al., 2014; Cao et al., 2015) have shown DOR expression at both mRNA and protein levels in HEK293 cells. We have also found that DOR activation can modify microRNA expression in rat kidney under prolonged hypoxia (He et al., 2013b), suggesting an important role of DOR in renal adaptation to hypoxic stress.

We previously showed that DOR is highly expressed in cortical neurons (Ma et al., 2005) and plays a critical role in neuroprotection against hypoxic injury (Zhang et al., 2002). A severe and prolonged hypoxia (e.g., 1% oxygen for 72 h) largely reduced the level of DOR with severe injury in the cortical neurons (Ma et al., 2005). In sharp contrast, the same hypoxic stress only caused a mild cell injury in NRK-52E cells after long-term exposure as shown in our previous study (Luo et al., 2017) and this present work. This is likely attributed to, at least partially, the tolerance of DOR to the hypoxic stress in the rat kidney epithelial cells, since DOR remains the “normal” level in these cells after 24–72 h of hypoxic exposure as shown in this study. Indeed, the level of DOR expression is likely related to the tolerance of hypoxic stress because the high density of DOR in the turtle brain may make turtle neurons more tolerant to hypoxia than rat neurons (Xia et al., 1992; Xia and Haddad, 2001). Since hypoxia was not able to decrease the expression of DOR in the rat kidney epithelial cells, DOR may be more resistant to hypoxic stress in these renal cells than neurons, which may be one of the reasons that these kidney cells are more tolerant to hypoxic stress. The present data further suggest that DOR expression may be a critical determinant of renal cell survival during hypoxic stress and its activity may serve as a protective factor against hypoxic kidney injury.

The major differences between the kidney and neuronal cells in DOR expression and cell survival under prolonged hypoxia inspirited us to further explore the roles of DOR in the regulation of ERK1/2 and p38 in the kidney cells, since ERK1/2 and p38 are two critical and opposite signaling regulators in the DOR-mediated neuroprotection (Ma et al., 2005). A surprising finding is that both ERK1/2 and p38 are seriously up-regulated in terms of their phosphorylation by hypoxic stress, while both of them are largely suppressed by DOR activation in NRK-52E cells. This is very different from our observation on neuronal cells in which DOR activation by hypoxic preconditioning attenuated the increase in P-p38 and partially reversed the decrease in P-ERK1/2 in severe hypoxia, suggesting different mechanisms in the DOR-mediated regulation of MAPKs between neuronal and renal cells.

In view of the fact that most of previous studies showing a harmful role of p38 (Li et al., 2016; Liu W.Y. et al., 2017) and protective role of ERK1/2 (Hung et al., 2003; Chen et al., 2015; Li et al., 2018) in cell survival under stress, the up-regulation of P-ERK1/2 in hypoxic renal cells is probably an adaptive response to overcome the hypoxia-induced increase in P-p38, rendering the cells a new balance between ERK1/2 and p38. Indeed, there is solid evidence indicating that P-ERK1/2 is a mediator of controlling cell survival in response to many stimuli including hypoxia in the kidney (di Mari et al., 1999; Kunduzova et al., 2002; Yang et al., 2003; Zou et al., 2016), while the p38 is considered as the major stress-activated protein kinases (Kyriakis and Avruch, 2012) and mediate a pro-inflammation effect in hypoxic condition (Wu et al., 2016). Based on our previous work, we believe that DOR, which was tolerant to hypoxia and was not down-regulated in the renal cells after hypoxia, plays a positive effect in the upstream signaling of P-ERK1/2 and a negative influence on p38, at least partially. On the other hand, DOR plays its protective role via multiple mechanisms (Ma et al., 2005; Chao and Xia, 2010; He et al., 2013a; Xia, 2015). When strengthening DOR signaling by adding DOR agonists, other mechanisms might be activated, which leads to a reduction in the hypoxia-induced increase in P-p38. Therefore, ERK1/2 is accordingly adjusted for the economy of cellular energy resources. Moreover, p38 is likely sensitive to DOR and DOR inhibition reverses the DOR-induced reduction of p38 phosphorylation because the level of P-p38 was high after the treatment with both DOR agonist and antagonist. The differential responses to DOR inhibition between p38 and ERK1/2 further suggest that there is a DOR-mediated mechanism(s) that affects p38 more than ERK1/2 in these cells. However, it is unknown why P-p38 was enhanced by UFP-512 or/and naltrindole treatment in normoxic conditions. It seems that p38 responds to DOR activation differently in hypoxia and normaxia.

Tumor necrosis factor-α is a potent pro-inflammatory molecule and can trigger downstream signaling cascades that control numerous cellular processes linked to cell viability and gene expression (Park and Bowers, 2010; Watters and O’Connor, 2011; Liu and McCullough, 2013). Our previous work showed that exogenous addition of TNF-α to the culture media worsened the hypoxia-induced damage in PC-12 cells (Wang et al., 2014). The present work further demonstrates that TNF-α can reduce the level of P-ERK1/2, suggesting that its insult to cell survival may be partially via down-regulating ERK1/2 signaling.

A large number of experimental data have confirmed that MAPK signal pathways are closely related to the regulation of anti-inflammatory cytokines, such as Bcl-6, TTP, and IL-10 (Pantoja-Escobar et al., 2019; Ronkina et al., 2019; Zhang et al., 2019) although there are still controversies with some showing an inhibitory effect of MAPKs on certain anti-inflammatory cytokines in BV2 cell line (Lee et al., 2015) and others having different opinions in bone marrow cells and microglial cells (Chi et al., 2006; Correa et al., 2010). In the kidney or renal cells, however, there is a lack of information on the relationship between MAPKs and anti-inflammatory cytokines in hypoxic condition. Our present study represents the first investigation into the relationship between MAPKs and anti-inflammatory cytokines and demonstrated that Bcl-6 is sensitive to hypoxic stress and DOR activation in the renal cells.

Bcl-6 protein, a transcriptional repressor, is a critical regulator of germinal centers and involves in the development and progression of cancers with uncertain mechanism (Basso and Dalla-Favera, 2010; Yu et al., 2015) and is relevant to MAPK signaling process (Batlle et al., 2009). Our previous study showed that it was down-regulated after hypoxic exposure, in parallel with an up-regulation of both ERK1/2 and p38 MAPKs (Luo et al., 2017). An intriguing finding in the present work is that the expression of Bcl-6 is negatively correlated with the phosphorylation of MAPKs and modulated by DOR function. For example, when the levels of P-ERK1/2 and P-p38 were down-regulated by DOR activation, the level of Bcl-6 was increased after 48 h exposure to hypoxia. The same is true in normoxia. This is the first work to show the regulation of DOR in Bcl-6 expression via MAPKs in kidney epithelial cells. There is evidence suggesting that MAPKs can regulate the hypoxic exposure level of Bcl-6 by a rapid degradation through the ubiquitin/proteasome pathway (Niu et al., 1998). Therefore, the DOR-MAPK mechanisms may play a role in the regulation of Bcl-6 through ubiquitination and proteasomal degradation pathway. However, the downstream signaling in the interaction between MAPKs and Bcl-6 in kidney epithelial cells is not well acknowledged yet.

Tristetraprolin is an mRNA-destabilizing and RNA-binding protein and can enhance the decay of mRNAs, and plays as an anti-inflammatory cytokine (Patial and Blackshear, 2016). TTP can limit the expression of a number of critical genes for chronic inflammatory diseases (Brooks and Blackshear, 2013; Prabhala and Ammit, 2015). Some studies suggested that the RNA degradation activity of TTP is negatively regulated by p38 MAPK-dependent signaling (Stoecklin et al., 2004; Brook et al., 2006; Hitti et al., 2006). However, the impact of the p38 pathway on TTP expression and function remains controversial (O’Neil et al., 2018). TTP is found at sites of inflammation, and is co-localized with active p38 MAPK (Ross et al., 2017). It is implied that the strong expression of TTP protein may function as a suppressor of inflammation by down-regulating inflammatory cytokines, chemokines, as well as adhesion molecules (Bollmann et al., 2014). Based on the present work, TTP is insensitive to hypoxic stress, but it increased to other severe insults such as DOR inhibition and TNF-α exposure. We propose that the enhanced TTP expression may devote to combat cellular injury caused by DOR inhibition or TNF-α inflammation.

**FIGURE 9:**
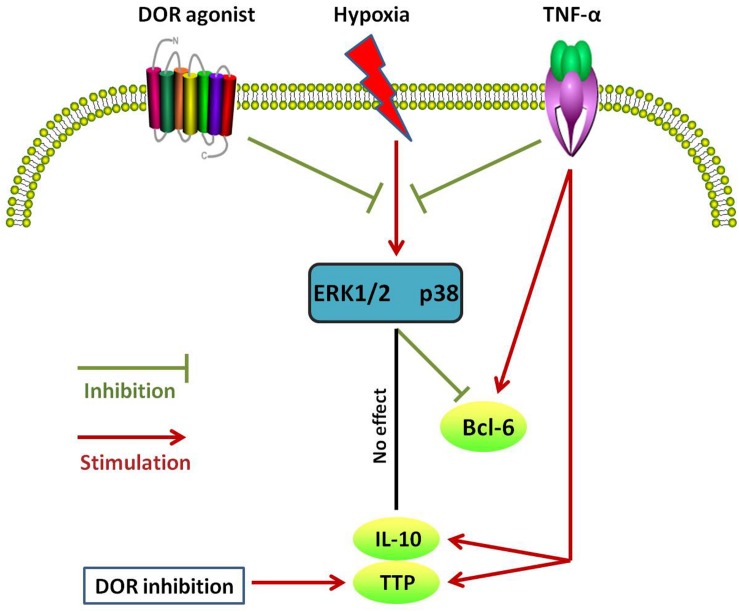
Schematic diagram of different changes in Bcl-6, TTP, and IL-10 expression in response to DOR activation, hypoxia, and TNF-α in NRK-52E cells. Note that these anti-inflammatory cytokines differentially response to different cell treatments.

Interleukin-10 is produced by many kinds of cells including T and B cells as well as epithelial cells (Williams et al., 2004). It inhibits transcription of a subset of pro-inflammatory genes (Denys et al., 2002; Murray, 2005). However, the mechanisms underlying IL-10-mediated anti-inflammatory responses are not well known. Here we firstly report that DOR is a regulator of such anti-inflammatory mechanism because DOR activation increases the level of IL-10 in normaxia. Interestingly, hypoxia alone could not induce any changes in IL-10 expression (Luo et al., 2017) and the same was true for DOR activation under hypoxic condition. It seems that IL-10 is more sensitive to DOR activation in normoxia than hypoxic condition. The major results of the present work are summarized in Table 1 and Figure 9. In brief, our data suggest that DOR may upregulate Bcl-6 via targeting MAPKs in rat kidney epithelial cells under hypoxia. In contrast, TTP and IL-10 are insensitive to DOR activation in hypoxia. Our data may provide a clue to the development of new therapeutic strategies for the prevention or/and treatment of hypoxic kidney injury as may occur in a range of clinically important conditions including ischemia–reperfusion injury and chronic kidney diseases.

## Data Availability Statement

All datasets generated for this study are included in the article and original records are available upon request.

## Ethics Statement

This study was carried out in accordance with the recommendations of the Ethics Committee of Soochow University.

## Author Contributions

FL and YX designed this study. FL and GS performed the experiments. FL and HL analyzed the results. RX coordinated the project. XH provided the research reagents. FL, XH, and YX wrote the manuscript. All authors approved the final version of manuscript.

## Conflict of Interest

The authors declare that the research was conducted in the absence of any commercial or financial relationships that could be construed as a potential conflict of interest.
